# Calretinin Participates in Regulating Steroidogenesis by PLC-Ca^2+^-PKC Pathway in Leydig Cells

**DOI:** 10.1038/s41598-018-25427-3

**Published:** 2018-05-09

**Authors:** Wendan Xu, Qian Zhu, Shan Liu, Xiaonan Dai, Bei Zhang, Chao Gao, Li Gao, Jiayin Liu, Yugui Cui

**Affiliations:** 1 State Key Laboratory of Reproductive Medicine, Clinical Center of Reproductive Medicine, First Affiliated Hospital, Nanjing Medical University, Nanjing, 210029 China; 2 Center of Reproductive Medicine, Bethune International Peace Hospital, Hebei Shijiazhuang, China; 3 Nanjing Maternal and Child Care Service Center, Nanjing Medical University, Nanjing, 210005 China

## Abstract

Calretinin, a Ca^2+^-binding protein, participates in many cellular events. Our previous studies found the high expression of calretinin in testicular Leydig cells. In this study, (MLTC-1 cells were infected with LV-calb2, R2C cells with LV-siRNA-calb2. The primary mouse Leydig cells were also used to confirm those data from cell lines. Testosterone level was significantly higher in the MLTC-1 cells with over-expressed calretinin than in the control, while progesterone was lower in the R2C cells in which down-regulated calretinin. The expressions of StAR changed in synchrony with hormones. Cytoplasmic Ca^2+^ level was significantly increased when calretinin was over-expressed. When MLTC-1 cells were infected with LV-calb2 and then stimulated using Clopiazonic, a Ca^2+^-releasing agent, testosterone was significantly increased. Interestingly, the expression levels of PLC, p-PKCµ (PKD), p-MARCKS and CREB, were significantly increased in the MLTC-1 cells with over-expressed calretinin, while PLC, p-PKD, p-MARCKS, MARCKS and CREB were decreased in the R2C cells with down-regulated calretinin. We also observed the increased expression of calretinin up-regulated testosterone production and the expressions of StAR and PLC in primary mouse Leydig cells. So, calretinin as a Ca^2+^-binding protein participates in the regulation of steroidogenesis via the PLC-Ca^2+^-PKC pathway in Leydig cells.

## Introduction

The main functions of testis are spermatogenesis and androgen production. Androgenesis is completed in testicular Leydig cells. Androgen (mainly including testosterone and dihydrotestosterone) combining with androgen receptor (AR) plays important roles in promoting the male sexual differentiation and puberty development, and maintaining the secondary sex characteristics, sexual maturation and male fertility, etc^1^. Testosterone secreted by Leydig cells is the main androgen in male individuals. The synthesis and secretion of testosterone are classically controlled by the hypothalamus-pituitary-gonadal axis (HPG), where gonadotropin-releasing hormone (GnRH) is secreted in pulses by the hypothalamus. GnRH acts on the anterior pituitary gland via its portal venous system, to prompt the secretion of corpus luteum formation hormone (LH) and follicle-stimulating hormone (FSH) in the synchronous pulses^2^. LH combining with its receptor (LHR) of Leydig cells, activates the adenylate cyclase and converts the adenosine triphosphate (ATP) to cyclic adenosine monophosphate (cAMP). Then the protein kinase A (PKA) is activated by cAMP, phosphorylating those steroidogenetic enzymes or activating transcription factors to promote the expression of steroidogenetic enzymes, which enhances the synthesis of testosterone. This is the so-called classic LH-PKA signal pathway^3^. Besides, LH combining with its receptor, activates the phospholipase C (PLC), and then divides phosphatidylinositol (PIP2) into diacylglycerol (DAG) and inositol trisphosphate 3 (IP3), the latter then triggers the release of Ca^2+^ from the calcium pool of endoplasmic reticulum. The cytoplasmic calcium ions are increased together with DAG, and activate protein kinase C (PKC), by which phosphorylates steroidogenetic enzymes or activates transcription factors to promote the expression of steroidogenetic enzymes, such as the transcription factors CREB and STAR, leading to the promotion of testosterone synthesis^4^. So Ca^2+^ is also involved in the regulation of sex steroid synthesis as a second messenger. After binding with LHR, LH can also promote the increase of intracellular Ca^2+^ concentration via ryanodine receptor (RyR), and then increase the synthesis of sex hormones by PKC pathway or calmodulin CaM^5,6^. In addition to the endocrine regulation, androgen synthesis is also influenced by paracrine and autocrine, such as insulin-like growth factor and interleukin 1 (IL-1), etc^7^. Therefore, steroidogenesis in Leydig cells is regulated by the complex networks of endocrine, autocrine and paracrine factors.

Calcium retinal protein 2 (calretinin, calb2), a kind of calcium binding protein, is mainly expressed in the nervous system, also in the ovary, adrenal glands and testis^8–11^. The main effect of calretinin is as the buffer of intracellular calcium ion to prevent the overload of Ca^2+^. Also, it can work as the Ca^2+^ receptor^12^. Ca^2+^, as a second messenger in cytoplasm, participates in a variety of physiological functions, such as cell proliferation and apoptosis, smooth muscle cell contraction, the pass of neurotransmitter in nerve cells, etc^13–16^. As mentioned above, Ca^2+^ is also involved in the regulation of synthesis of sex hormones. Our preliminary studies showed that adult rats expressed highest calretinin in the cytoplasm of Leydig cells when compared with preadolescent or old rats, which is in accord with the androgen level, suggesting that calretinin may prompt the steroidogenesis by Ca^2+^-related pathway in Leydig cells^17^.

We assumed that the calretinin, as a local and intracellular factor, can promote steroidogenesis by the PLC-Ca^2+^-PKC pathway in Leydig cells. To test our hypothesis, we used two kinds of Leydig cell lines as the model by over-expression and down-expression of calretinin, to study subsequently the effects of calretinin on androgen production in Leydig cells and the underlying mechanism.

## Results

### Changes of calretinin expression in Leydig cells infected with Lv-calb2 and LV- siRNA-calb2

**Figure 1:**
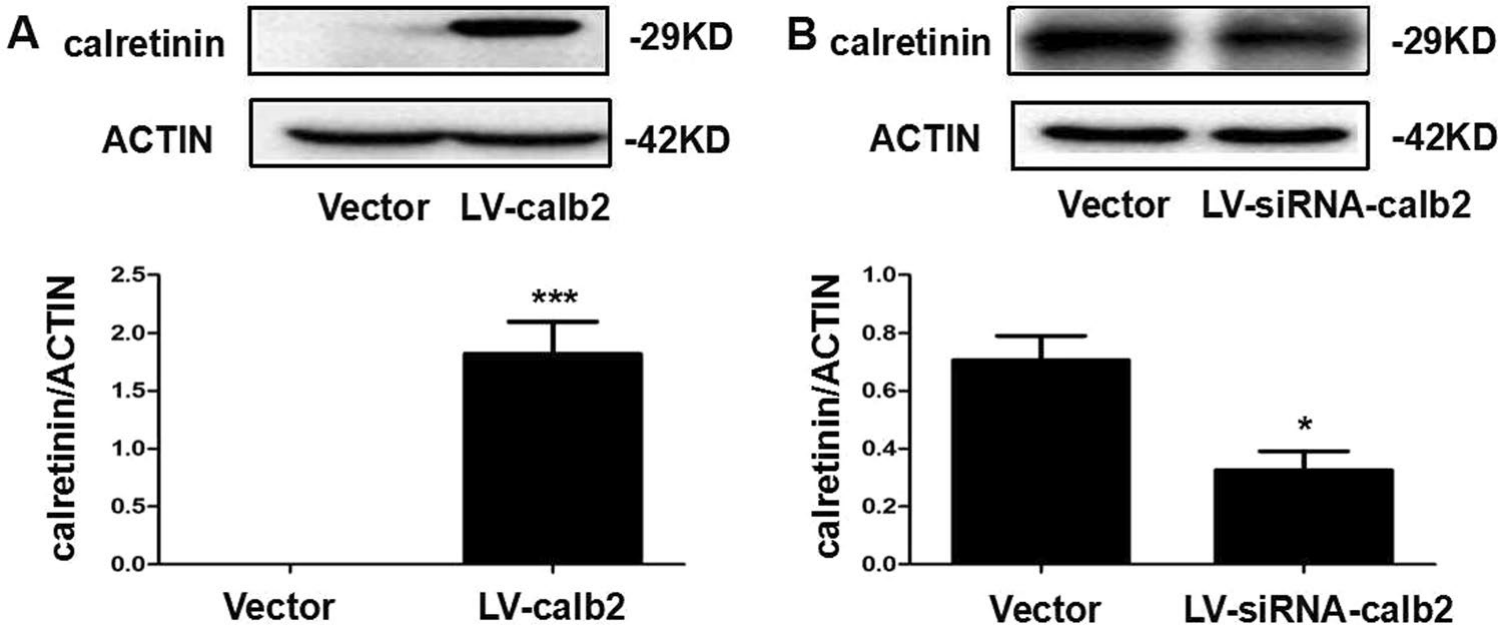
The expression of calretinin in the MLTC-1 and R2C cells infected with LV-calb2 and LV-siRNA-calb2. (**A**) The expression of calretinin in the MLTC-1 cells infected with LV-calb2. Compared with the vector infection group, the LV-calb2 infection cells had significantly higher expression of calretinin (*p* < 0.001). (**B**) The expression of calretinin in the R2C cells infected with LV-siRNA-calb2. Compared with the vector infection group, the LV-siRNA-calb2 infection cells had significantly lower expression of calretinin (*p* < 0.05). All experiments were independently repeated three times. *p < 0.05; ***p < 0.001. The original blot images can be found in Supplmental Fig. S1.

To investigate the potential effect of calretinin on steroidogenesis, MLTC-1 cell and R2C cell were infected by Lv-calb2 and LV-siRNA-calb2, respectively. After 96 h of infection, the expression of calretinin was measured by western blot. We found that the expression of calretinin was significantly up-regulated in the MLTC-1 cells after Lv-calb2 infection (*p* < 0.001) (Fig. 1A). In the R2C cell infected with LV-siRNA-calb2, the expression of calretinin was significantly down-regulated (*p* < 0.05), and the interference efficiency was up to 60% (Fig. 1B). Figure 1 showed that the LV-calb2 and LV-siRNA-calb2 lentiviral vectors were highly efficient.

**Figure 2:**
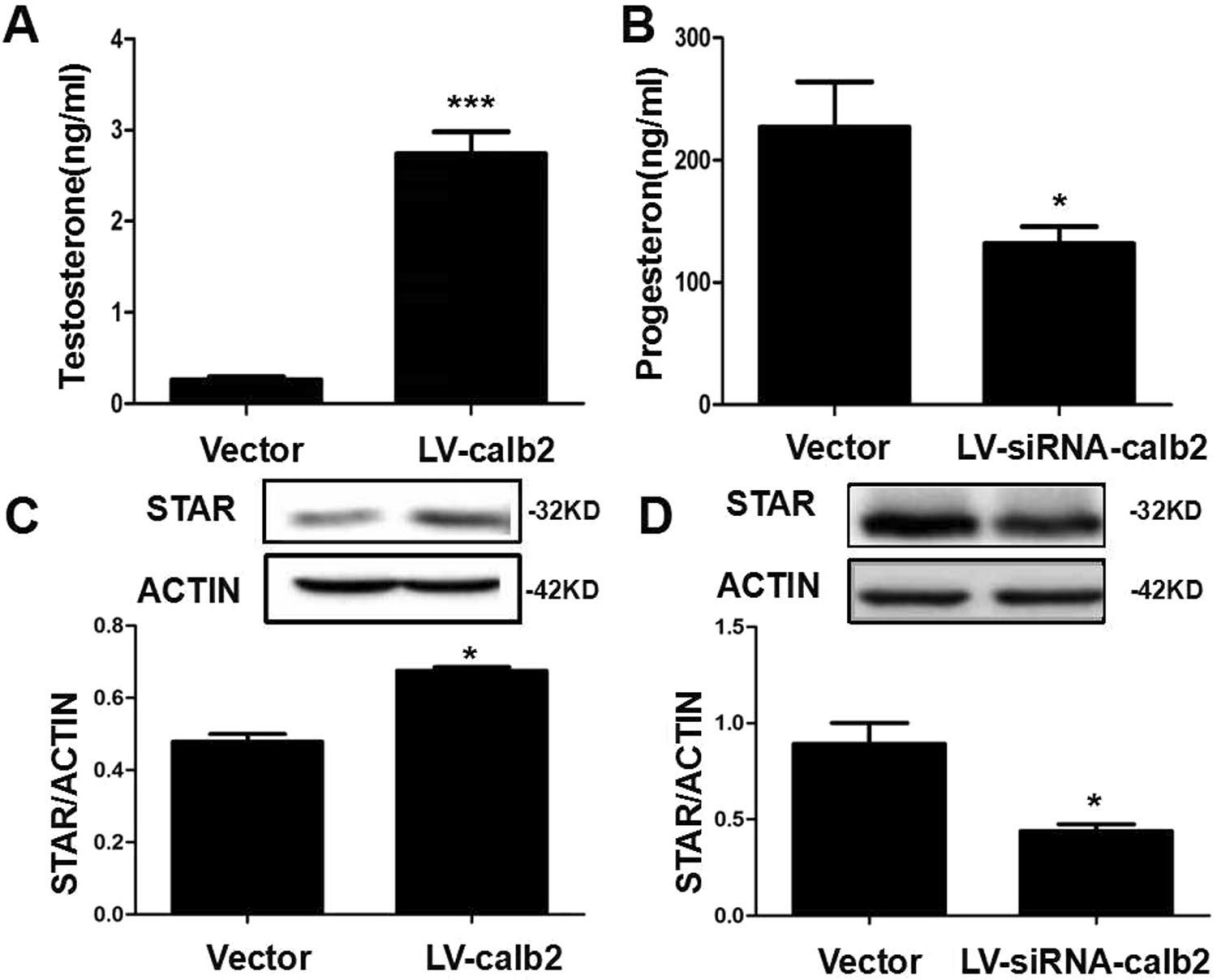
Hormonal levels in the culture medium and the expression of StAR in the Leydig cells infected with LV-calb2 and LV-siRNA-calb2. (**A**) The level of testosterone in the culture medium of MLTC-1 cells infected with LV-calb2 or vector. The testosterone secretion was significantly increased when calretinin was over-expressed in MLTC-1 cells (*p* < 0.001). (**B**) The level of progesterone in the culture medium of R2C cells infected with LV-siRNA-calb2 or vector. The progesterone secretion was significantly decreased when the calretinin expression was down-regulated in R2C cells (*p* < 0.05). (**C**) The expression of StAR in the MLTC-1 cells infected with LV-calb2 or vector. The expression level of StAR was significantly increased when calretinin was over-expressed in MLTC-1cells (*p* < 0.05). (**D**) The expression of StAR in the R2C cells infected with LV-siRNA-calb2 or vector. The expression level of StAR was significantly decreased when the calretinin expression was down-regulated in R2C cells (*p* < 0.05). All experiments were repeated three times. *p < 0.05; ***p < 0.001. The original blot images can be found in Supplemental Fig. S2.

The levels of testosterone and progesterone in the culture supernatant were assessed. The results showed that after 96 h of infection, the testosterone level in the culture supernatant of MLTC-1 cells with LV-calb2 infection was significantly increased (*p* < 0.001, Fig. 2A), while the progesterone level in the culture supernatant of R2C cells with LV-siRNA-calb2 infection was significantly decreased (*p* < 0.05, Fig. 2B). And we found that the change of StAR expression was similar to the change of steroid hormones in cells with LV-calb2 or LV-siRNA-calb2 infection. When the expression of calretinin was up-regulated, the StAR expression was also increased in MLTC-1 cells (*p* < 0.05, Fig. 2C); while the expression of calretinin was down-regulated, the StAR expression was lowered in R2C cells (*p* < 0.05, Fig. 2D).

### The expression of PKC signal pathway molecules

**Figure 3:**
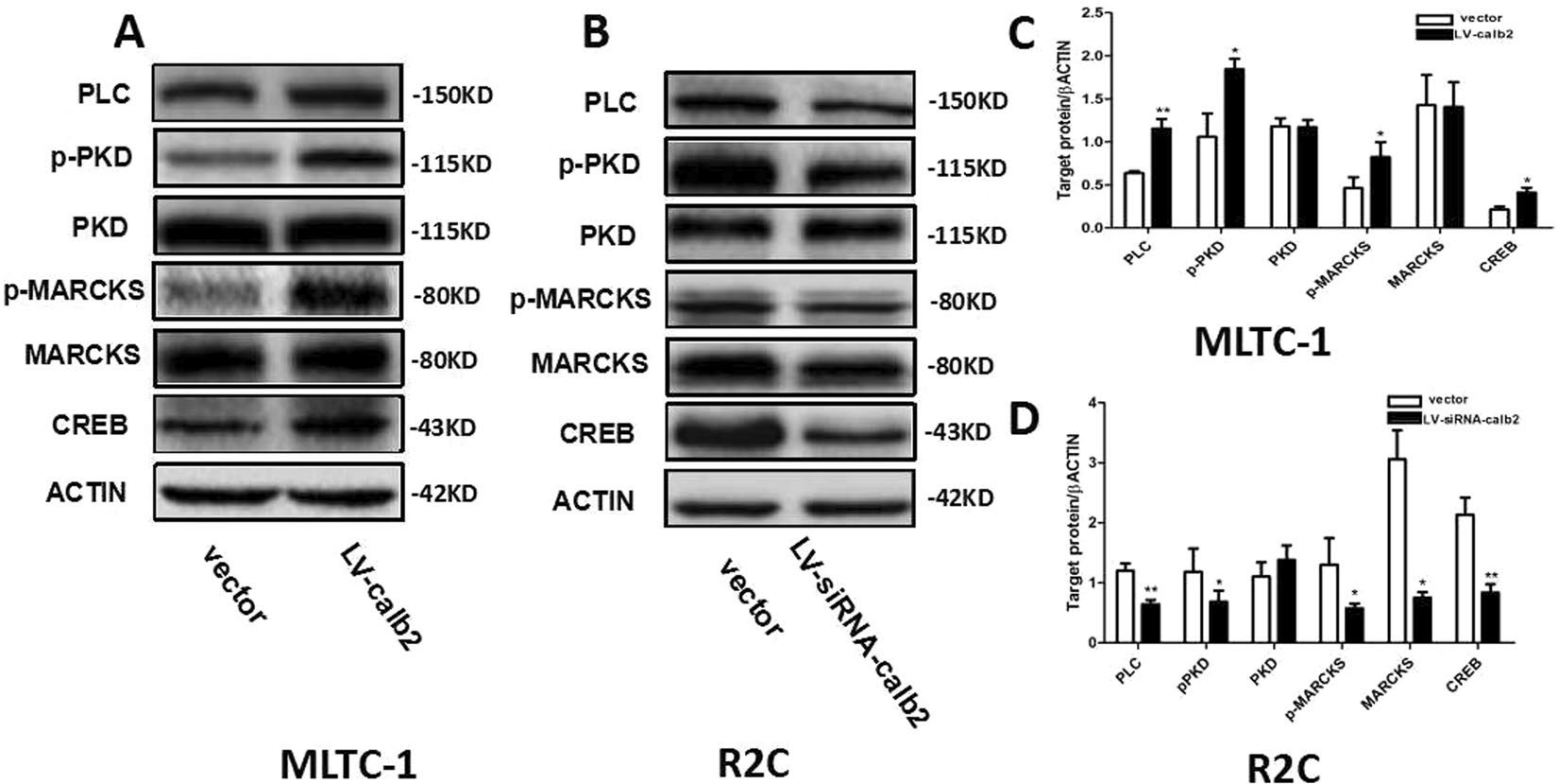
Expression of those factors in the PKC pathway in the Leydig cells after the overexpression and downregulation of calretinin. (**A**,**C**) The expression levels of PLC, p-PKD, PKD, p-MARCKS, MARCKS and CREB in the MLTC-1 cells infected with LV-calb2 or vector. The expression levels of PLC, p-PKD, p-MARCKS, and CREB were significantly increased in the MLTC-1 cells with over-expression of calretinin, when compared with the control group (*p* < 0.05 or 0.01)). The changes of total MARCKS and PKD were not significant (*p* > 0.05). (**B** and **D**) The expression levels of PLC, p-PKD, PKD, p-MARCKS, MARCKS and CREB in the R2C cells infected with LV-siRNA-calb2 or vector. The expression levels of PLC, p-PKD, p-MARCKS, MARCKS and CREB were significantly decreased in the R2C cells with down-expression of calretinin, when compared with the control group (*p* < 0.05 or 0.01)). The change of total PKD was not significant (*p* > 0.05). All experiments were independently repeated three times. *p < 0.05; **p < 0.01. The original blot images can be found in Supplemental Fig. S3-4.

To explore the potential mechanism of calretinin action, the expression of those molecules in PKC signal pathway was observed by western blot, including PLC, p-PKD, p-MARCKS, MARCKS and CREB (Fig. 3). As mentioned above, MLTC-1 cells with the full set of testosterone synthetases can synthesize and secretion testosterone. We measured the level of testosterone to evaluate androgen systhesis. However, the expression level of calretinin is very low in MLTC-1 cells (Fig. 1A). MLTC-1 cells were used as the cell model of calretinin over-expression to explore the potential mechanism of calretinin action. R2C cells can synthesize progesterone, not testosterone, because of the lack of CYP17A1. Therefore, the level of progesterone was measured to evaluate steroidogenesis. Basal expression of calretinin in R2C cells was high (Fig. 1B), so R2C cells were used as the cell model of calretinin down-regulation. We found thatin the MLTC-1 cells infected by LV-calb2, the expressions of PLC, p-PKD, p-MARCKS, and CREB were significantly increased (*p* < 0.05 or 0.01) and the expressions of MARCKS and PKD did not change (*p* > 0.05) (Fig. 3A,C). In the R2C cells infected by LV-siRNA-calb2, the expressions of PLC, p-PKD, p-MARCKS, MARCKS and CREB were significantly reduced (*p* < 0.05 or 0.01) and the expression of PKD did not change (*p* > 0.05, Fig. 3D).

### Ca^2+^ regulates steroidogenesis in Leydig cells

**Figure 4:**
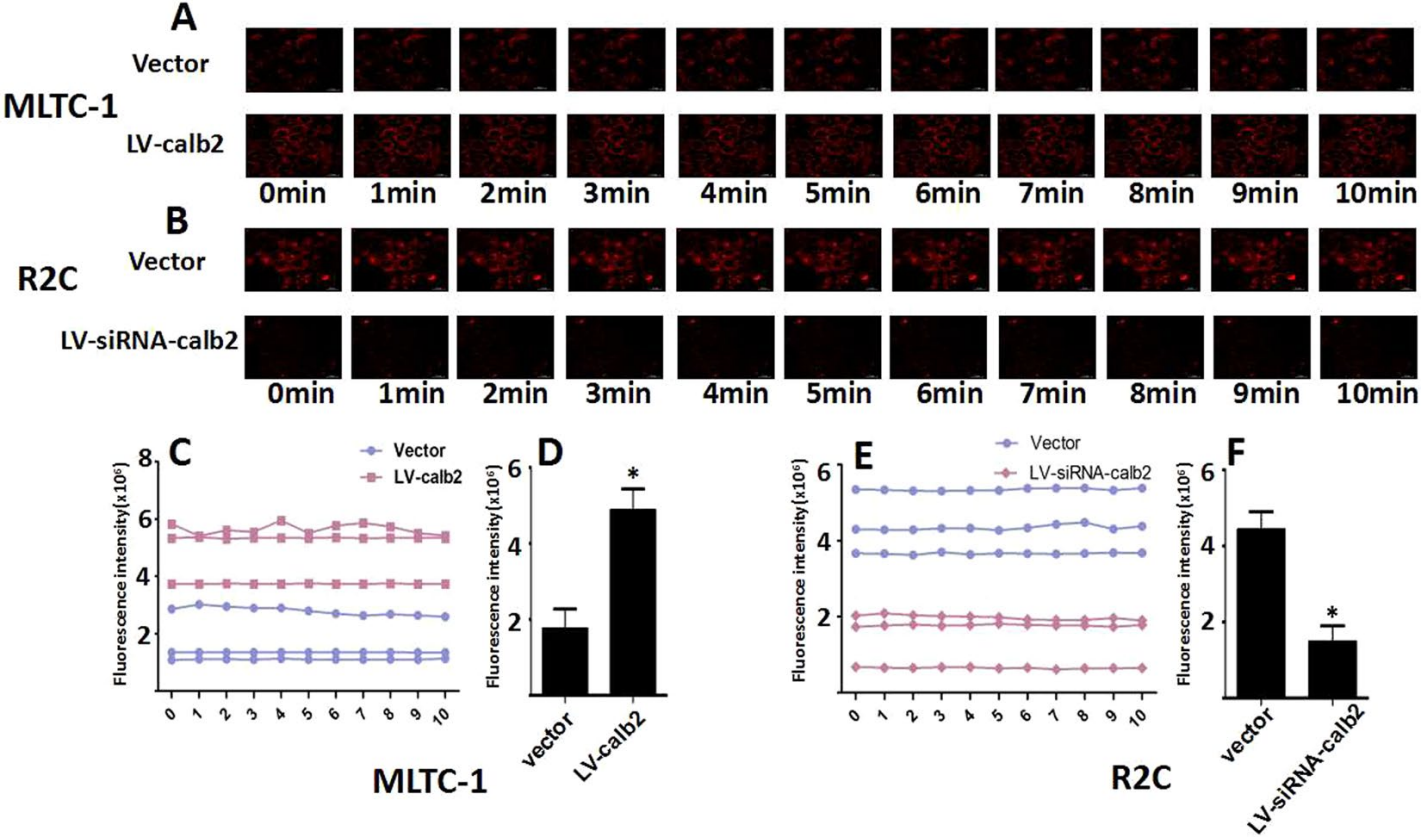
Changes of Cytoplasmic Ca^2+^ in the MLTC-1 and R2C cells after LV-calb2 or LV-siRNA-calb2 infection. The Ca^2+^ level was measured using X-Rhod-1 Calcium Imaging Kit. Intracellular Ca^2+^ was probed using X-Rhod-1 and images were captured every 1 min for 10 min. There was no visible change of signal strength in single cells during the time course, All experiments were repeated for three times. (**A**,**C**) The change of fluorescence intensity in the MLTC-1 cells infected with LV-calb2 or vector. The basal signal of Ca^2+^ in the LV-calb2 infected MLTC-1 cells was significantly stronger (*p* < 0.05), but the fluorescence intensity was not differently changed during 10 minutes of time-course. (**B** and **E**) The change of fluorescence intensity in the R2C cells infected with LV-siRNA-calb2 or vector. The basal signal of Ca^2+^ in the LV-siRNA-calb2 infected R2C cells was weaker (*p* < 0.05), but the fluorescence intensity was not differently changed during 10 minutes of time-course. (**D** and **F**) The signal intensities of those cells infected with LV-calb2 or LV-siRNA-calb2 at the time-point of 5 min were respectively compared with their controls. The intensity was significantly increased by the over-expression of calretinin in MLTC-1 cells (D, *p* < 0.05), while the intensity was decreased by the down-expression of calretinin in R2C cells (F, *p* < 0.05). *p < 0.05.

To explore whether calcium activates PKC, Ca^2+^ changes in the two cell lines after LV-calb2 or LV-siRNA-calb2 infection were detected (Fig. 4). The basal signal of Ca^2+^ in the LV-calb2 infected MLTC-1 cells was significantly stronger than that in vector infected MLTC-1 cells (Fig. 4A,C, *p* < 0.05). In the R2C cells infected with LV-siRNA-calb2, the basal signal of Ca^2+^ was weaker when compared with that of the vector transfected cells (Fig. 4B,D, *p* < 0.05). The MLTC-1 and R2C cells infected respectively with LV-calb2 and LV-siRNA-calb2 for 96 h were then incubated in medium with X-Rhod-1AM for 30 min at 37 °C in the dark. The X-Rhod-1 was excited at 561 nm and emission signal was collected at 594 nm. This excited time point was set as 0 min of time-course.The fluorescence intensity was not differently changed during 10 minutes of time-course. And there was no visible change of signal strength in single cell during the time-course. The result of time-course suggested that the Ca^2+^ oscillation did not occur. Therefore, the signal intensities of those cells infected with LV-calb2 or LV-siRNA-calb2 at the time-point of 5 min were respectively compared with their controls. The intensity was significantly increased by the over-expression of calretinin in MLTC-1 cells, while the intensity was decreased by the down-expression of calretinin in R2C cells (Fig. 4D,F, both *p* < 0.05).

**Figure 5:**
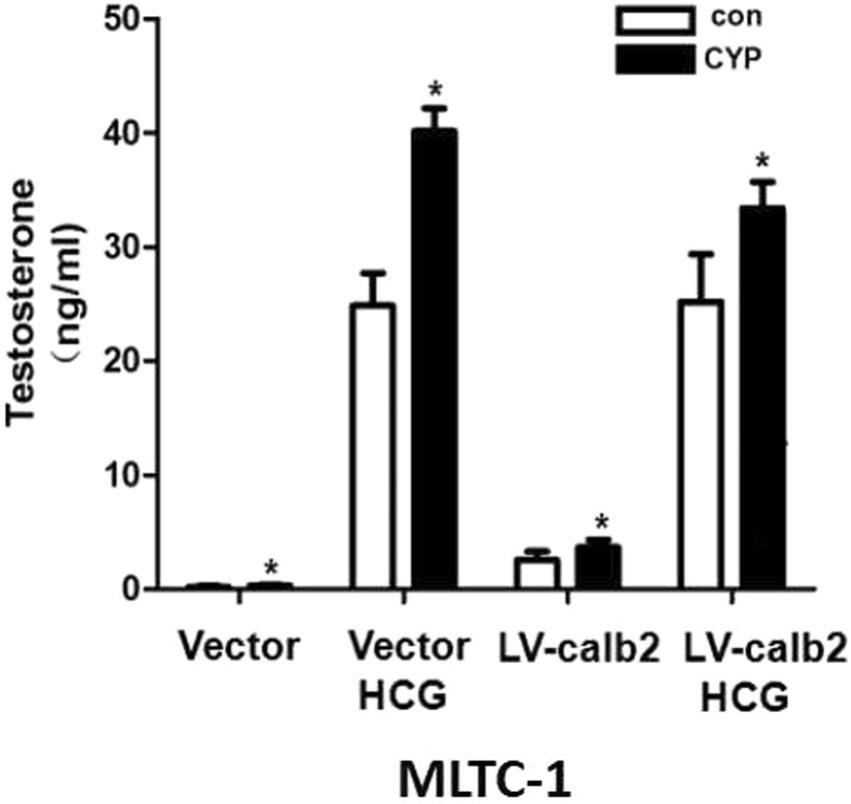
Testosterone secretion in the MLTC-1 cells infected with LV-calb2 or vector when treated by hCG and Ca2+ releasing agent CYP. The testosterone production was significantly increased by hCG treatment. the testosterone production in the LV-calb2 infected MLTC-1 cells after hCG treatment was not significantly higher than that in the vector infected cells. After the vector or LV-calb2 infected MLTC-1 cells were stimulated by CYP, the testosterone production was significantly increased regardless of the presence of hCG treatment, All experiments were independently repeated three times. *p < 0.05.

What relationship was between hCG treatment and calretinin as well as Ca^2+^? After MLTC-1 cells were treated with hCG (0.01–0.4 U/ml) for 4 h, the culture supernatant was collected for the testosterone assay. Results showed that the testosterone production was significantly increased by hCG in a dose-dependent manner. After the vector or LV-calb2 infected MLTC-1 cells were stimulated by CYP, the Ca^2+^ releasing agent, the testosterone production was significantly increased regardless of the presence of hCG treatment (Fig. 5, all *p* < 0.05). Even so, the testosterone production in the LV-calb2 infected MLTC-1 cells after hCG treatment was not significantly higher than that in the vector infected cells (*p* > 0.05). The results indicated that Ca^2+^ participated in the regulation of steroidogenesis in Leydig cells.

### The Increased expression of calretinin up-regulated testosterone production, and StAR and PLC expression, in mouse primary Leydig cells

**Figure 6:**
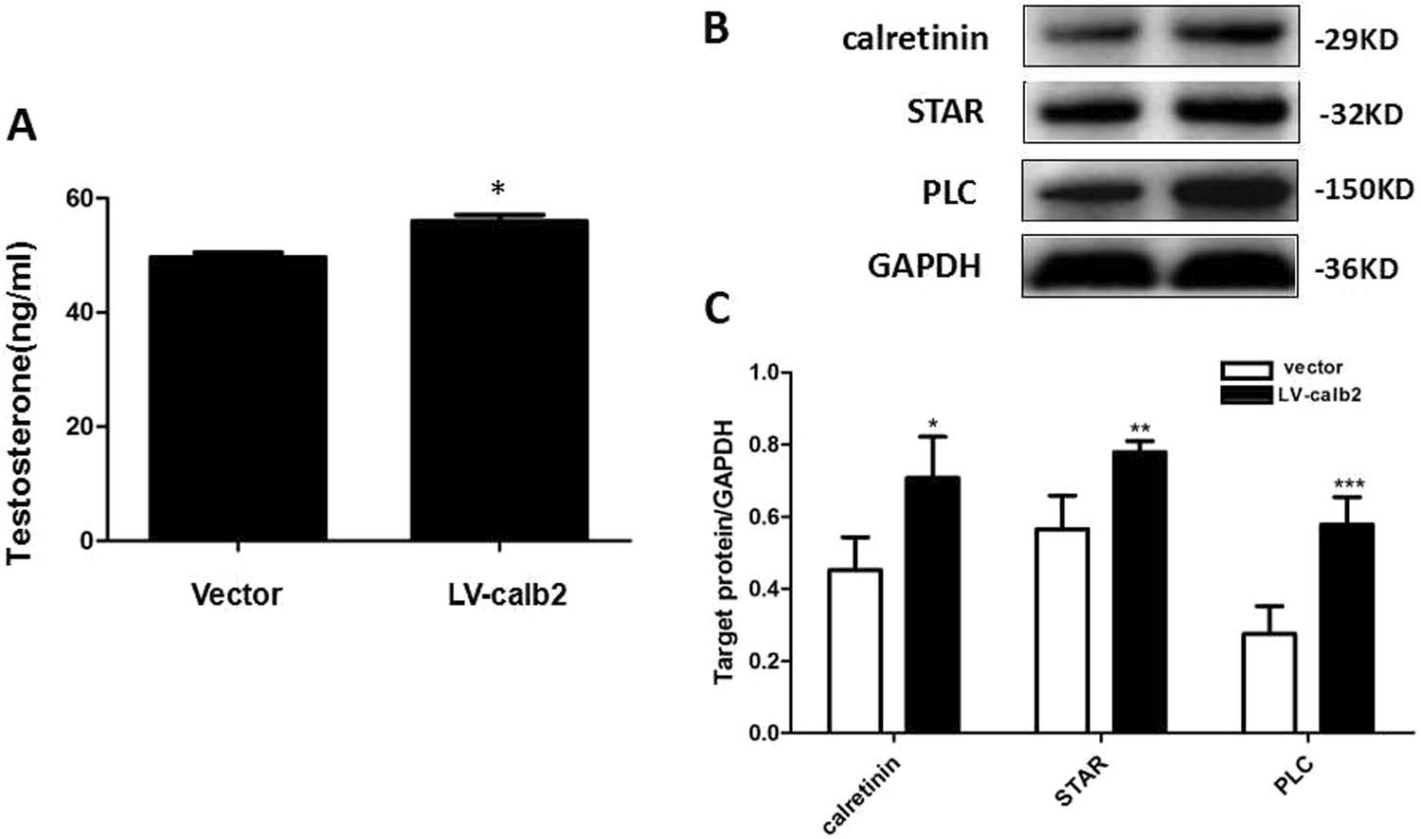
The testosterone secretion and expressions of StAR and PLC in the primary mouse Leydig cells transfected with LV-calb2 or vector. (**A**,**C**) Change of testosterone level in the culture medium of the primary mouse Leydig cells transfected with LV-calb2 or vector. The level of testosterone was significantly increased in the LV-calb2 infected cells (*p* < 0.01). (**B**,**C**) The expressions of calretinin, StAR and PLC in the primary mouse Leydig cells transfected with LV-calb2 or vector. The expression of StAR was significantly increased when calretinin was up-regulated ((*p* < 0.01). Meanwhile, the expression of PLC was significantly increased in the primary mouse Leydig cells transfected with LV-calb2 ((*p* < 0.001). All experiments were independently repeated three times. *p < 0.05; **p < 0.01; ***p < 0.001. The original blot images can be found in Supplemental Fig. S5.

The expressions of calretinin, StAR and PLC proteins were examined in the mouse primary Leydig cells infected with LV-calb2, while the testosterone level in the culture supernatant was measured (Fig. 6). The western blotting showed a stronger band of calretinin in the LV-calb2 transfected samples, along with StAR and PLC (Fig. 6B,C, *p* < 0.05 or *p* < 0.01), no visible change was shown in GAPDH, whild the testosterone level was significantly increased (Fig. 6A, *p* < 0.01). The results were consistent with those data from MLTC-1 and R2C cells.

## Discussion

The main function of Leydig cells is androgen synthesis. The main raw material of androgen synthesis is cholesterol, which from either the ester of plasma lipoproteins, or directly from the extracellular cholesterol into Leydig cells cytoplasm through scavenger receptor (SR-B1). StAR will transfer cholesterol from the outer mitochondrial membrane into the inner membrane where cholesterol side-chain lyase (CYP11A1) resides. Regulation of androgen synthesis is mostly implemented by regulating the synthesis or phosphorylation of StAR, one of the key rate-limiting gate of androgen production^7^.

**Figure 7:**
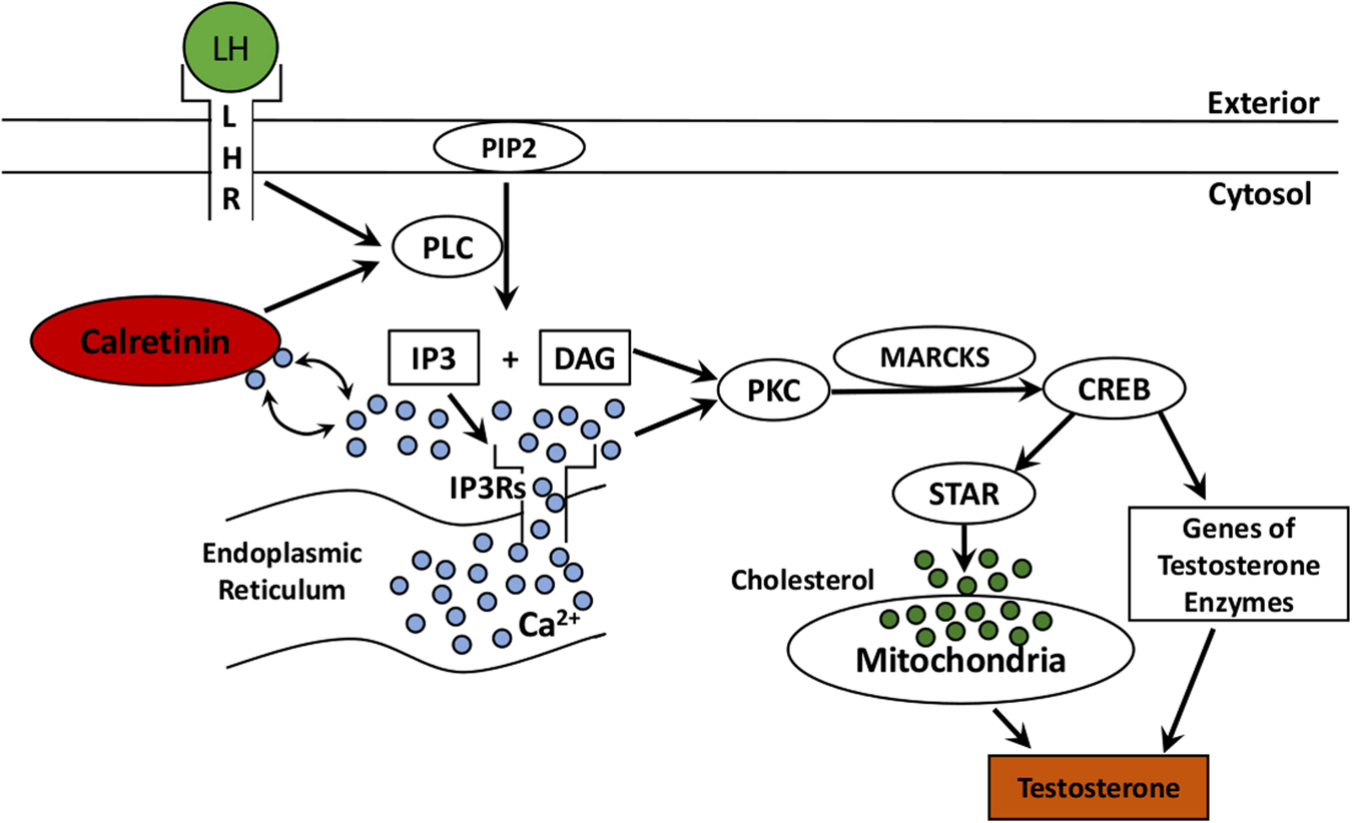
Calretinin participates in the regulation of steroidogenesis by PLC-Ca^2+^-PKC pathway in Leydig cells. LH/LHR activates PLC, and PLC then divides PIP2 into γDAG and IP3. IP3 can activate Ca^2+^ channels in endoplasmic reticulum. Ca^2+^ together with DAG activates PKC, which phosphorylates MARCKS to promote the expressions of steroidogenetic enzymes, including StAR. Calretinin can buffer free Ca^2+^ and regulate the level of intracellular Ca^2+^ through the PLC-Ca^2+^-PKC pathway. Ca^2+^, as a second messenger, activates PKC, followed by the phosphorylation of MARCKS.

Calretinin, a Ca^2+^ binding protein, is believed to function as a sensor and buffer of intracellular Ca^2+^ to prevent the Ca^2+^ overloading. More and more evidence indicates that calretinin involves in multiple physiological process by regulating Ca^2+ ^^12^. In human, calretinin can be detected in Leydig cells through fetal and adult life^18,19^. The level of calretinin in human fetal testis is correlated with the number of Leydig cells, strongly implying that it may be associated with steroidogenesis^18^. Our previous study also raised calretinin as the local factor in the regulation of steroidogenesis in Leydig cells^17^. Herein, our present study provided further evidence that calretinin can modulate the production of both testosterone and progesterone. We found that in rodent Leydig cells, the level of intracellular free Ca^2+^, as well as the steroidogenesis, changes along with the expression of calretinin. During this process, the expressions of those factors involved in the PLC-Ca^2+^-PKC pathway also change and finally affect the expression of StAR. This effect occurs not only in two Leydig cell lines but also in the primary mouse Leydig cells. We summarized roughly the action of calretinin and potential mechanism (Fig. 7). The StAR phosphorylation and the levels of many steroidogenic enzymes will be measured in our furture study to evaluate furtherly the activation of androgen production in Leydig cells.

Studies have shown that Ca^2+^, as a second messenger of LH pathway, regulates steroidogenesis in Leydig cells besides the classical LH-PKA pathway^20–25^. LH promotes the synthesis and secretion of testosterone in Leydig cells by both PKA and PKC pathway. The increased level of Ca^2+^ in cytoplasm is required when LH stimulates the normal androgen synthesis^26^. Recent studies indicate that the intracellular free Ca^2+^ in Leydig cells is derived from both the extracellular Ca^2+^ via internal flow and the organelles, such as mitochondria and endoplasmic reticulum, via calcium outflow. When Leydig cells are stimulated by LH, the extracellular calcium flows into the cytoplasm through T-type Ca^2+^ channels on the cell membranes; and then through the T-type Ca^2+^ channels, Ca^2+^ is released from endoplasmic reticulum into the cytoplasm^27^. Endoplasmic reticulum in Leydig cells has two kinds of T-type calcium channels: RyRs and IP3 receptor (IP_3_Rs)^26^. Activation of RyRs is the main regulation of Ca^2+^ level, however how RyRs is activated by LH remains unclear. It is well known that LH can activate the PLC, crack PIP2 into IP3 and DAG^28^, as showed in Fig. 7. IP3 combining with IP3Rs can activate Ca^2+^ channels in endoplasmic reticulum^29^, so as to increase cytoplasmic level of Ca^2+^. Calretinin in the cytoplasm can bind and buffer Ca^2+^ to maintain a relatively stable concentration of Ca^2+^ within the cytoplasm^12^. In our study, using calcium ion probe X-Rhod-1, we observed that the change of intracellular Ca^2+^ level parallels the calretinin level of Leydig cells (Figs 4 and 5), implying that calretinin not only acts as a buffer to prevent the overloading of Ca^2+^ but also recruits more free Ca^2+^ to cytoplasm. Our study demonstrated that PLC expression was up-regulated in LV-calb2 infected MLTC-1 and primary mouse Leydig cells, and down-regulated after calretinin was intervened in R2C cells (Figs 3 and 6). Therefore, besides direct binding and buffering, the calretinin may adjust the releasing of Ca^2+^ via PLC as well (Fig. 7). The modulated intracellular free Ca^2+^ is then further involved in steroidogenesis. It is worthy to notice that despite the calretinin expression highly increased the intracellular Ca^2+^ level in MLTC-1 cells, the testosterone secretion did not show a dramatic change on the basis of hCG treatment (Fig. 5). The MLTC-1 cells are derived from mouse Leydig cell tumor and therefore show some unique characteristic compared to normal Leydig cells. It was reported that MLTC-1 produced the low level of testosterone without hCH stimualtion^20^, just like our result (Fig. 2, the vector transfected MLTC-1 cells), and that MLTC-1 transcripted *17β-HSD* mRNA isoform 7 instead of isoform 3 when stimulated by hCG^21^. The major function of 17β-HSD isoform 7 is to convert the estrone to estradiol^22^ and the cholesterol biosynthesis^23^, therefore its function on testosterone production is different. This potentially explains the low synthesis of testosterone in MLTC-1 cells. Even if StAR expression was increased by calretinin or Ca^2+^ and more cholesterol was transported to mitochondria, the downstream testosterone synthesis was still limited without hCG stimulation.

The increased free Ca^2+^ can promote steroidogenesis in Leydig cells^30^. A study also suggested that the maintenance of mitochondria Ca^2+^ concentration was necessary to support steroidogenesis in Leydig cells^31^. PKC can be directly activated by calcium and DAG, and then promote the phosphorylation of its specific substrate, MARCKS, to increase the expression of transcription factor, CREB (as shown in Fig. 7). CREB activates the expression of StAR, and promote steroidogenesis in Leydig cells in the end^6^. Our present study found that the expression change of StAR was consistent to the change of the steroid hormones after the calretinin expression was up-regulated or interrupted in Leydig cells (Fig. 2). Furthermore, the factors involved in the Ca^2+^-PKC pathway, including p-PKD, p-MARCKS and CREB, were increased when the expression of calretinin was increased, while those factors such as p-PKD, p-MARCKS, MARCKS and CREB were decreased when calretinin was down-regulated. The results suggest that the StAR expression is regulated by calretinin through Ca^2+^-PKC pathway.

Our results also suggested that the testosterone production was increased after the vector or LV-calb2 infected MLTC-1 cells were stimulated by CYP regardless of the presence of hCG (Fig. 5). CYP, a Ca^2+^ releasing reagent, specifically inhibits the Ca^2+^-ATPase on sarcoplasm/endoplasmic reticulum but does not affect the binding between calretinin and Ca^2+^. Adding CYP to the MLTC-1 cells will cause a rapid increase of free Ca^2+^ in cytoplasm, in terms of which calretinin will act as the Ca^2+^-exclusive buffer to maintain the relative stability of Ca^2+^, rather than continuously promote it. Under the abnormal Ca^2+^ releasing, calretinin may help to retrieve the balance of cytosolic free Ca^2+^, and partly mask its effect on steroidogenesis. We hypotheses that the lower increase of testosterone in the *LV-calb2* infected cells with hCG/CYP double treatment (when compared to the vector cells) revealed a potential two-way adjustment of calretinin to excessive free Ca^2+^. Those cells with excessive [Ca^2+^]i induced by hCG/CYP double treatment were caught in the depressed state of steroidogenesis. This hypothesis needs to be studied furtherly in future.

The level of testosterone or progesterone in the culture medium was measured in this study. Strictly, we did not directly measure the androgen synthesis. Ca^2+^ can also stimulate the release of vesicles, therefore it is difficult to distinguish directly the synthesis or secretion. However, our results could be mainly related to the androgen synthesis. Those factors involved in the PLC-Ca^2+^-PKC pathway were decreased in the R2C cells with down-regulated calretinin, while the StAR expression was decreased as well. Ca^2+^ did not only affect the secretion of vesicles but also affect the transport of cholesterol. Also as shown in Fig. 5, when MLTC-1 cells were treated by CYP (which should highly increase the intracellular Ca^2+^ level), the secretion of testosterone in the vector transfected MLTC-1 cells was still not so high as that in the LV-calb2 transfected cells. It could be PLC besides Ca^2+^, however most current researches on calretinin were focused on its interaction with Ca^2+^. There is no further clue about what the other pathway might be involed.

Our results are roughly summarized in Fig. 7. Calretinin can buffer free Ca^2+^ and regulate the level of intracellular Ca^2+^ through the PLC-Ca^2+^-PKC pathway. Ca^2+^, as a second messenger, activates PKC, followed by the phosphorylation of MARCKS. The p-MARCKSs increase the expression of the transcription factor CREB, which will promote the expression of StAR and other steroidogenesis enzymes. StAR further transports more cholesterol into mitochondria and finally results in the increased androgen synthesis in Leydig cells^6^. Therefore, the PLC-Ca^2+^-PKC signal pathway was involved in the mechanism of the calretinin regulated steroidogenesis in Leydig cells. It needs more study to understand whether other Ca^2+^-related pathways, such as the extracellular Ca^2+^ via internal flow and PKA pathway, are also involved in this regulation.

Late-onset gonad hypofunction syndrome (LOH) occurs in 13% of 40 to 49 years old males and 30% of 50 to 59 years old males^32^, which causes the symptoms such as decreased libido, depression, infertility, androgen reduction-related cardiovascular disease, increased incidence rate of bone fractures and mortality in patients^33^. The core mechanism of LOH onset is the decreased testosterone level related with the decline of Leydig cell function^34^. Studies found that the age-related testosterone decrease is not associated with serum LH levels but the drop of LHR levels. Other related intracellular factors include cut of cyclic adenosine enzyme expression, increased number of cAMP decomposing enzymes, restrain of cholesterol synthesis and mobilization, and cut of hormone synthesis enzyme (CYP11A1, CYP17A1, StAR, 3β-HSD) in Leydig cells^35^. Some evidence also links the change to oxide balance disorders^36^. Clinical treatment tends to directly use external testosterone, which improves the quality of life, but will increase the risks of prostate cancer and red blood cell proliferation. Additionally, excessive exogenous androgen will also inhibit secretion of LH and FSH, and suppress spermatogenesis by negative feedback. Recently the idea of increasing testicular testosterone without disrupting the normal LH functions is advocated^37^. The intracellular calretinin and related PLC-Ca^2+^-PKC signal pathway could provide a potential biological treatment for LOH.

In conclusion, this research proves that calretinin participates in the regulation of steroidogenesis in Leydig cells, and also discussed the intracellular signal mechanism of steroidogenesis in Leydig cells. The potential significance is to remedy for male gonadal dysfunction diseases such as LOH.

## Methods

### Cell lines

In the present study, two kinds of cell lines were used. MLTC-1 cell, the mouse Leydig tumor cell line, can synthesis testosterone with the full set of testosterone synthetase, which can be regulated by hCG via LHR on cell membranes. MLTC-1 cell was purchased from the Cell Institute of Shanghai (Shanghai, China) and incubated in Roswell Park Institute-1640 medium (RPMI-1640; Hyclon, State of Utah, USA) supplemented with 10% fetal bovine serum (FBS, Gibco, Rockville, MD,USA), in the presence of 100 U/ml penicillin, and 100 g/ml streptomycin. In this study, we found that calretinin was expressed at very low level in MLTC-1 cells, which means that MLTC-1 is suitable to use as the cell model of calretinin over-expression so as to observe the change of testosterone.

R2C cell, the rat Leydig tumor cell line, was obtained from the ATCC (Manassas, VA, USA). R2C cell can synthesize progesterone. However, it cannot synthesize testosterone eventually because of the lack of 17α-hydroxylase/17, 20 lyase (CYP17A1)^30^. R2C cells were maintained in DMEM-F12 medium (Gibco, Rockville, MD, USA) supplemented with 15% horse serum (Gibco, Rockville, MD, USA) and 2.5% fetal bovine serum. All cells were maintained at 37 °C in an atmosphere of 5% CO_2_. We also found that calretinin was highly expressed in R2C, which means that R2C cell is suitable to use as the cell model of calretinin down-regulation to observe the change of progesterone.

### Reagents

The Ca^2+^ probe X-Rhod-1 was purchased from Molecular Probes. Anti-calretinin, anti-PLC, anti-PKD, and anti-MARCKS were obtained from Santa Cruz (Texas, USA). anti-p-PKD, anti-p-MARCKS and anti-StAR were acquired from Cell signaling Technology (Beverly MA,USA), anti-CREB was obtained from Proteintech Group (Chicago,USA), anti-ACTIN was bought from Sigma (Darmstadt, Germany), and anti-GAPDH was obtained from Bioworld Technology, Inc (Minnesota,USA). Anti-goat-horseradish peroxidase (HRP)-conjugated, anti-rabbit-HRP conjugated or anti-mouse-HRP conjugated secondary antibodies were purchased from Jackson immunoresearch company (PA, USA). Lentiviral vector system plasmid GV287 was bought from Stratagene company (CA, USA). T293, DH5α cells were provided by our laboratory. Reverse transcription polymerase chain reaction (RT-PCR) kits, restriction enzyme (BamHI/AgeI), T4 DNA ligase and DNA polymerase were bought from the NEB company (New England, USA). Plasmid extraction kits, gel recovery kits and PCR product recovery kits were bought from QIAGEN company (New York, USA). Lipofectamine TM2000 liposome transfection kits were bought from Invitrogen company (New York, USA). Trizol was obtained from Gibco company (New York, USA). Cyclopiazonic acid (CYP) was bought from Sigma (Darmstadt, Germany). RIA kits were purchased from Beijing North Institute of Biological Technology (Beijing, China). The Bicinchoninic Acid (BCA) kits, pancreatic enzyme, cell lysis buffer, protease inhibitors, phosphorylation protein inhibitors were bought from Beyotime (Shanghai, China). The ECL reagent was bought from TAKARA (Shiga, Japan).

### Isolation and primary culture of mouse Leydig cells

In this study, main data from MLTC-1 and R2C cell lines were confirmed in the primary mouse Leydig cells. Male C57BL/6J mice (4–6 weeks) were purchased from Vital River Laboratory Animal Technology (Beijing, China). All the experiments were carried out in accordance with the guidelines for the care and use of laboratory animals approved by the Animal Care Committee of Nanjing Medical University. Mice were sacrificed by cervical dislocation, and testes were dissected. Testes were washed twice in PBS. The connective tissues and tunica albuginea were then removed, and the pair of testes were incubated in a 50 ml tube with 5 mL 0.25% (w/v) collagenase I (Sigma) at 37 °C for 30 min, rotating at 1000 rpm. The digestion was stopped by adding 20 ml DMEM-F12 containing 10% FBS to the mixture. The supernatant was then filtered with 400 mesh stainless screen, and centrifuged at 1500 rpm for 5 min to pellet the cells. After washing with PBS twice, the cells were finally suspended and *in vitro* cultured in DMEM-F12 medium (Gibco) with 10% FBS (Gibco) at 37 °C, 5% CO_2_.

### Construction of lentiviral vectors

Primers were designed using the calb2 sequence in GenBank (GenBank accession number: NM_053988). The upstream primer was 5′-GGGTCAATATGTAATTTTC AGTG-3′, the downstream primer was 3′-CTGGTCGAGCTGGACGGCGACG-5′. Both of them contained AgeI and BamH I restriction enzyme sites. The calb2 gene was cloned by polymerase chain reaction (RT-PCR) using the following reaction conditions: 94 °C for 5 min; 94 °C for 30 s, 55 °C for 30 s, 72 °C extension for 2 min for 30 cycles; 72 °C extension for 10 min. The PCR products were purified, sequenced and cloned into the lentiviral over-expression vectors.

RNA interference fragments were designed according to the calb2 sequence (GenBank: NM_053988), and then calb2 RNAi effective target genes were screened. After the Oligo DNA of target sequences was synthesized (5′-CCGGCAGGAAGAG CGTCATGTCCTTCTCGAGAAGGACATGACGCTCTTCCTGTTTTTG-3′ and 5′- AATTCAAAAACAGGAAGAGCGTCATGTCCTTCTCGAGAAGGACATGACGCTCTTCCTG-3), the lentiviral vectors carrying LV-calb2-RNAi-EGFP (RNAi group) and LV-calb2-NC-EGFP (NC group) were constructed and packaged to produce the lentivirus venom.

### Cell infection

Because MLTC-1 expressed low level of calretinin and R2C highly expressed calretinin, MLTC-1 and R2C were respectively transfected with LV-calb2 and LV-siRNA-calb2 according to the MOI value of 100 and the titer of 1 × 10^4^ to over-express or down-express calretinin, respectively. MLTC-1 cell or R2C cell was cultured in each well of 12-well plate, and when cell density reached 30%, the LV-calb2 or LV-siRNA-calb2 virus was used to infect cells. The empty vector virus was also used as control. The final concentration of polybrene was 5 μl/ml. After 96 h, fluorescence was observed by microscope, total protein of cells was harvested for the protein imprinting (western Blot). The culture supernatant was collected, stored at −80 °C for testosterone or progesterone assay.

### Western blot

After the MLTC-1 and R2C cells infected with LV-calb2 and LV-siRNA-calb2 respectively, protein was exacted by Radioimmunoprecipitation (RIPA) lysis buffer. The protein concentration was detected by BCA kits following manufacturer’s instruction. Protein was loaded on 12% gel 50 µg per well, separated by SDS-PAGE and transferred to polyvinylidene difluoride (PVDF) membranes. Membranes were blocked, then probed with specific anti-calretinin (1:1000), anti-PLC (1:1000), anti-p-PKCµ (1:500), anti-PKD (1:500), anti-p-MARCKS (1:500), anti-MARCKS (1:1000), anti-CREB (1:500), anti-StAR (1:1000), anti-ACTIN (1:10000) antibodies at 4 °C, for overnight. After incubated with HRP-conjugated secondary antibodies at room temperature for 1 h, an enhanced chemiluminescence (ECL) kit was used for detecting the signals. The bands were semi-quantified with the analysis software provided by the imaging system.

### Radioimmunoassay

MLTC-1 cells were incubated and treated with different concentrations of hCG for 4 h. Then the culture supernatant was collected for the testosterone assay with the radioimmunoassay kits according to manufacturer’s instructions. After the MLTC-1 and R2C cells were infected with LV-calb2 and LV-siRNA-calb2 respectively, the culture supernatant was collected for the testosterone and progesterone assay. And after infected by vector and LV-calb2, MLTC-1 cells were stimulated by CYP with or without hCG for 4 h, followed by accumulation of the supernatant for the testosterone assay. Measurement sensitivities of testosterone and progesterone were 0.02 ng/ml and 0.2 ng/ml, respectively. The measurement precisions of testosterone and progestin: both inter-assay coefficients of variation were <10% and both intra-assay coefficients of variation were <15%.

### Recording Calcium level

The Ca^2+^ level was measured using X-Rhod-1 Calcium Imaging Kit following manufacturer’s instruction. Briefly, X-Rhod-1AM powder was dissolved in DMSO to make the 10 mM stock. After infected with LV-calb2 and LV-siRNA-calb2 respectively, MLTC-1 and R2C cells were washed by non-serum medium for twice. Then 10 μM X-Rhod-1AM in culture medium was added to the dishes. After incubated for 30 min at 37 °C in the dark, cells were washed with non-serum medium for twice again. Live-cell images were then captured using Nikon C2 Confocal Microscope. X-Rhod-1 was excited at 561 nm and emission signal was collected at 594 nm. We performed the time-course for both short and long terms in pre-experiments, from 1 ms per photo for 30 s (the exposure time still took 0.5 s) to 5 min per photo for 30 min. For each treatment, images of a single vision were taken every 1 min for 10 min. The fluorescence strength of each cell was semi-quantified by the Nikon Element AR software. Ten random cells in each photo were semi-quantified, and the mean of fluorescence strength was calculated. Those cells would be traced in the following time-course (0 to 10 min). All experiments were independently repeated three times. The tendency chart of the mean calcium from each photo was then plotted.

### Statistical methods

The SPSS 17.0 software was used for statistical analysis. All data were presented as mean ± standard deviation (Means ± SD). The single-factor samples were compared using independent sample *t* test. P < 0.05 was represented significantly different.

## Electronic supplementary material

Supp Info File
