# Supplementary material for: Calretinin Participates in Regulating Steroidogenesis by PLC-Ca2+-PKC Pathway in Leydig Cells
Source: Sci Rep. 2018 May 9;8:7403. doi: 10.1038/s41598-018-25427-3 (PMC5943404; doi:10.1038/s41598-018-25427-3)
Supplement: Supplementary file 1 — Supp Info File [file 41598_2018_25427_MOESM1_ESM.docx]

**Supplementary information**

Calretinin Participates in Regulating Steroidogenesis by PLC-Ca^2+^-PKC Pathway in Leydig Cells

Wendan Xu^1^, Qian Zhu^1^, Shan Liu^1,2^, Dai Xiaonan^1,3^, Bei Zhang^1^, Chao Gao^1^, Li Gao^1^, Jiayin Liu^1^, Yugui Cui^1*^

1 State Key Laboratory of Reproductive Medicine, Clinical Center of Reproductive Medicine, First Affiliated Hospital, Nanjing Medical University, Nanjing 210029, China.

2 Center of Reproductive Medicine, Bethune International Peace Hospital, Hebei Shijiazhuang, China.

3 Nanjing Maternal and Child Care Service Center, Nanjing Medical University, Nanjing 210005, China

**Supplementary figure S1.** The raw image of Figure 1A and B. Because of saving time and antibodies , the WB membrane for it and the next four supplementary figures in this experiment were been cut according to protein molecular weigh and protein marker to be incubated by antibaodies and exposured.


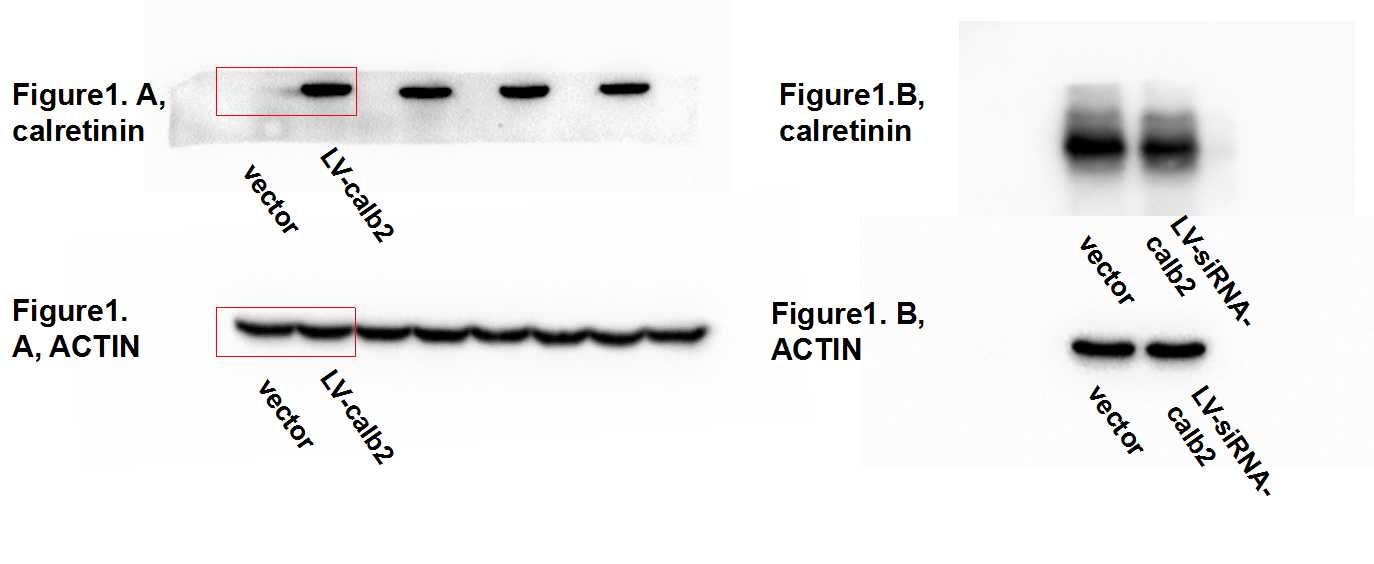


**Supplementary figure S2.** The raw image of Figure 2C and D.


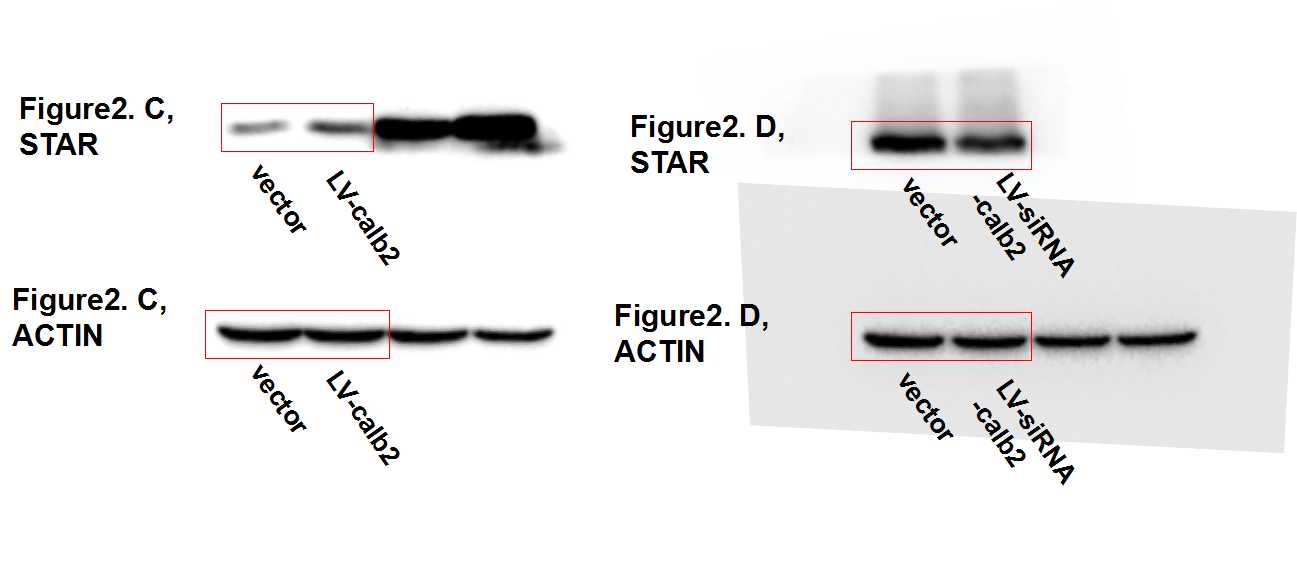


**Supplementary figure S3.** The raw image of Figure 3A.


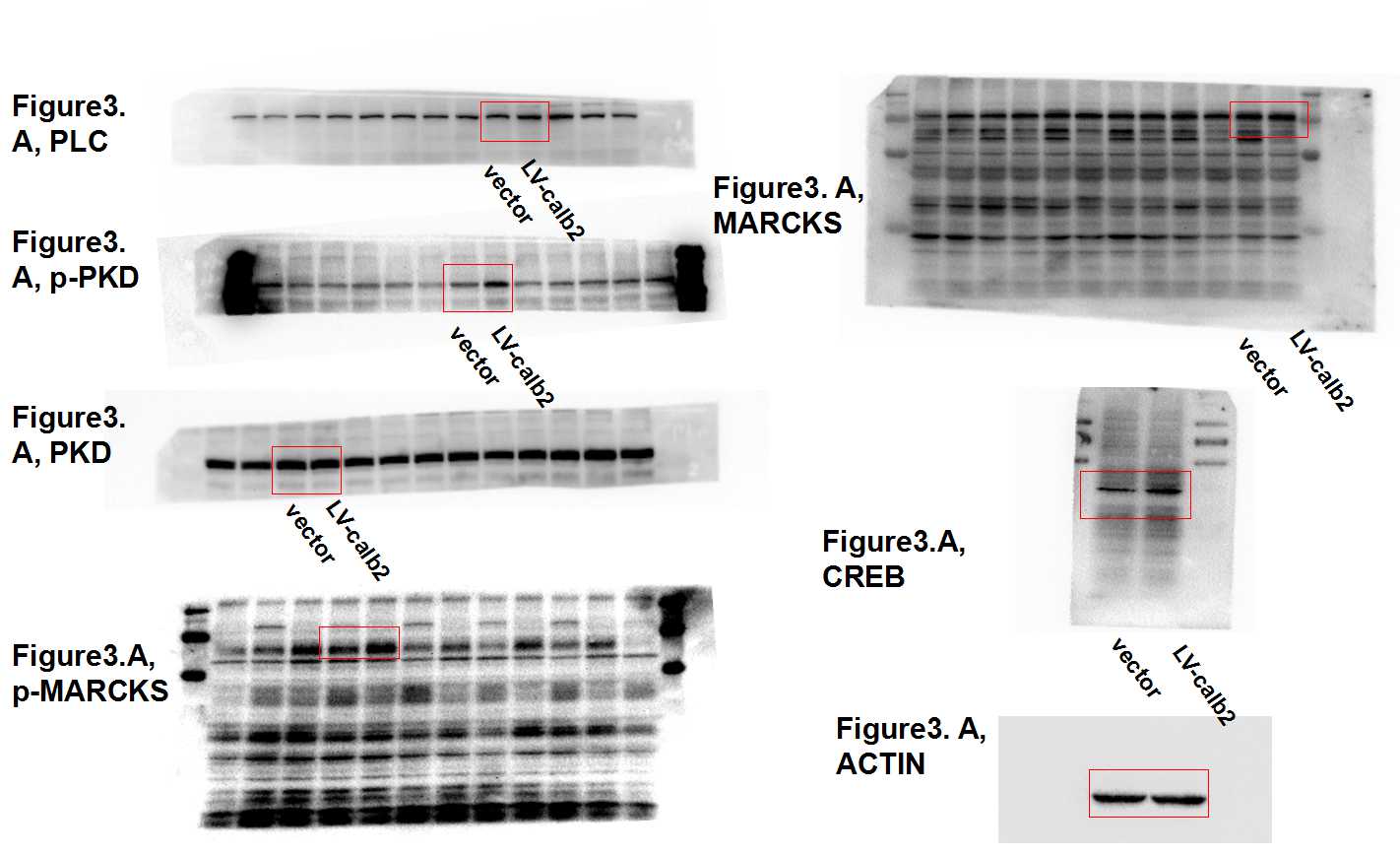


**Supplementary figure S4.** The raw image of Figure 3B.


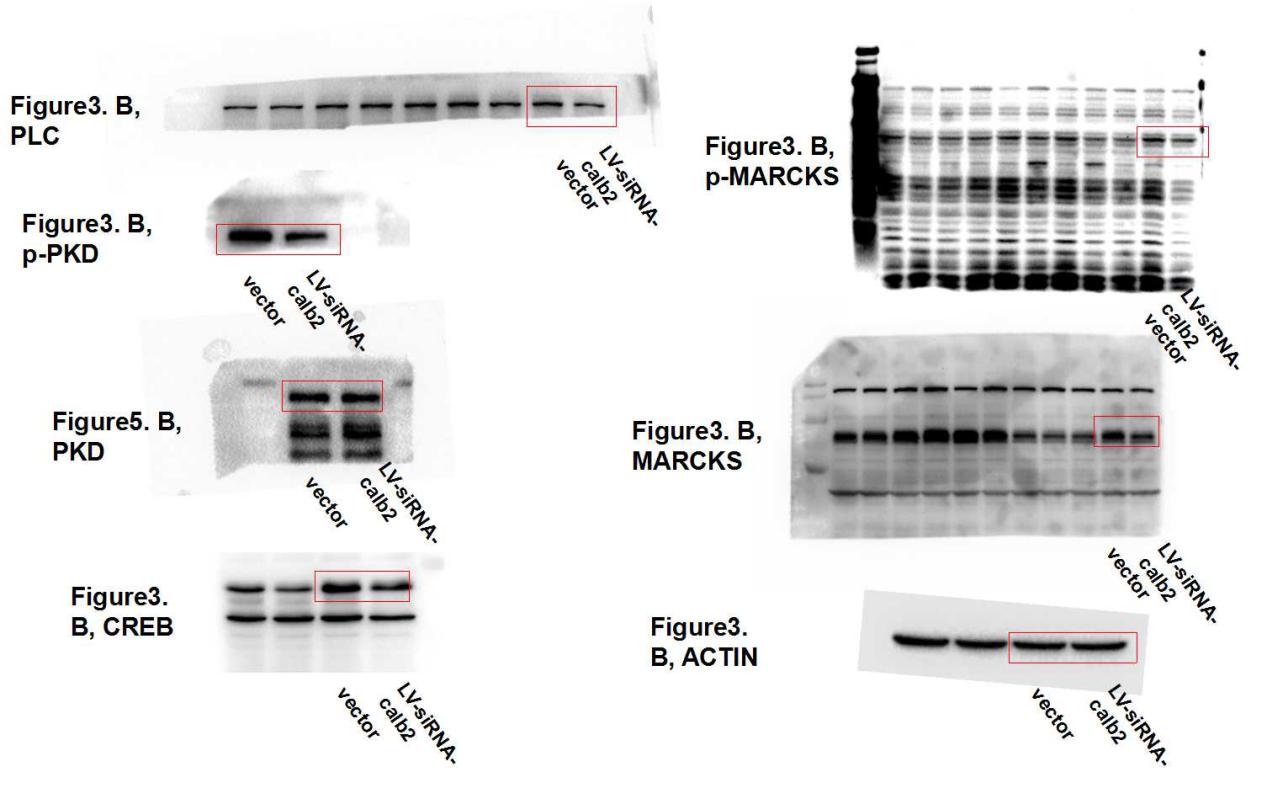


**Supplementary figure S5.** The raw image of Figure 6B.

**
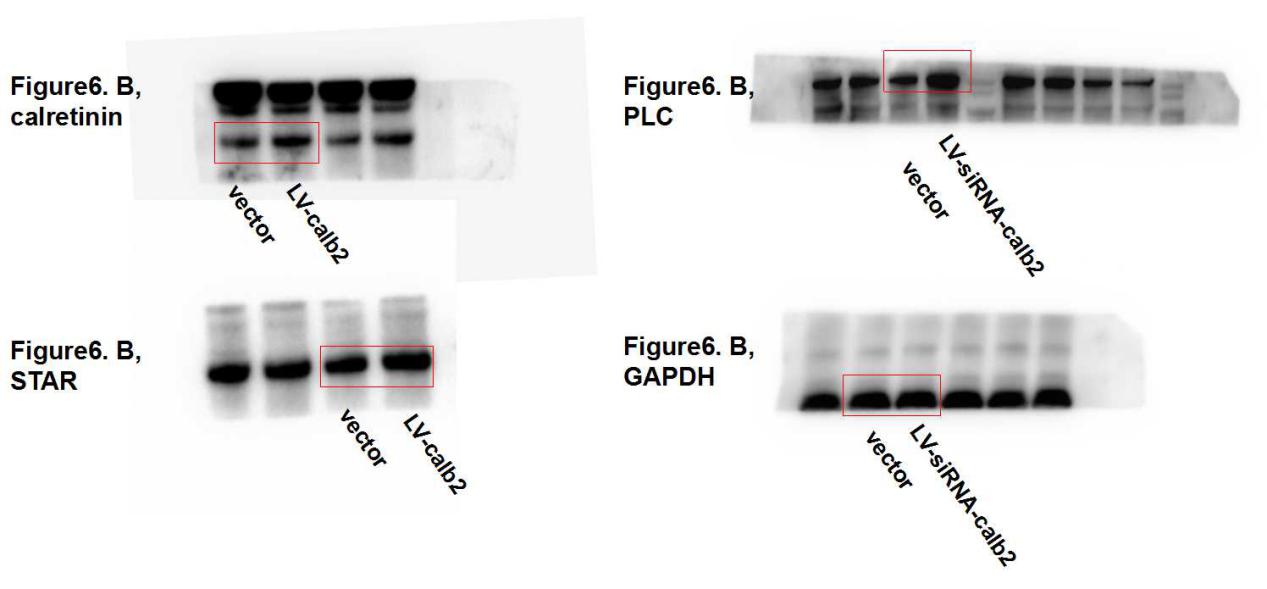
**
